# Supplementary material for: Inhibition of sterol O-acyltransferase 1 blocks Zika virus infection in cell lines and cerebral organoids
Source: Commun Biol. 2024 Sep 5;7:1089. doi: 10.1038/s42003-024-06776-4 (PMC11377701; doi:10.1038/s42003-024-06776-4)
Supplement: Supplementary file 2 — Supplemental Material [file 42003_2024_6776_MOESM2_ESM.pdf]

Inhibition of Sterol O-acyltransferase 1 Blocks Zika Virus Infection in Cell Lines and Cerebral Organoids

Supplementary Information

Anja Schöbel[1], Vinicius Pinho dos Reis[1], Rabea Burkhard[1], Julia Hehner [1], Laura Schneider[1], Martin Schauflinger[1], Gabrielle Vieyres[2], and Eva Herker[1*]

[1] Institute of Virology, Philipps-University Marburg, Marburg, Germany
[2] Institute of Virology and Cell Biology, University of Lübeck, Lübeck, Germany

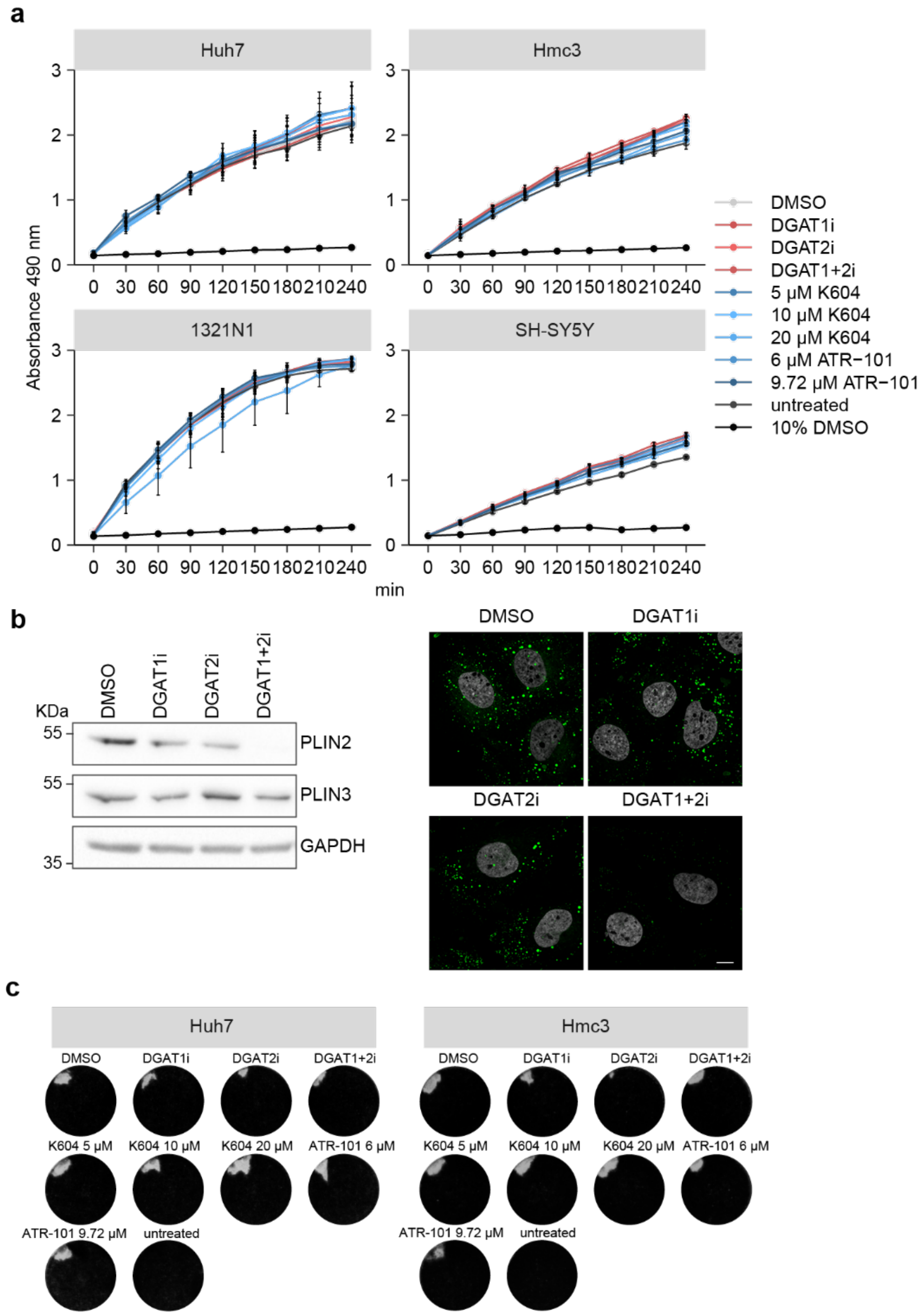

Supplementary Fig. 1: DGAT inhibitor and SOAT1 inhibitor treatment does not affect cell viability
(a) Cell viability assay of DGATi and SOAT1i-treated cell lines. Huh7, Hmc3, 1321N1, and SH-SY5Y cells were treated with the indicated inhibitor concentrations for 72 h before cell viability was measured. DMSO was used as vehicle control and 10% DMSO served as non-viable control (mean ± SEM, n = 2). (b) Functional validation of DGATi-treatment in Huh7 cells. Cells were treated with DGATi (5 µM each) or DMSO as vehicle control for 48 h. Cell lysates were analyzed by immunoblot using PLIN2 and PLIN3-specific antibodies. GAPDH served as loading control. For microscopy analysis, LDs were visualized with BODIPY493/503 (green), and nuclei were stained with Hoechst (grey) (scale bar 10 µm). Shown is one representative experiment. (c) Crystal violet staining of inhibitor-treated mock-infected Huh7 and Hmc3 cells. Cells were treated with the indicated DGAT and SOAT1 inhibitor concentrations for 96 h, fixed and cell monolayers were visualized with crystal violet Shown is one representative experiment (n = 2).

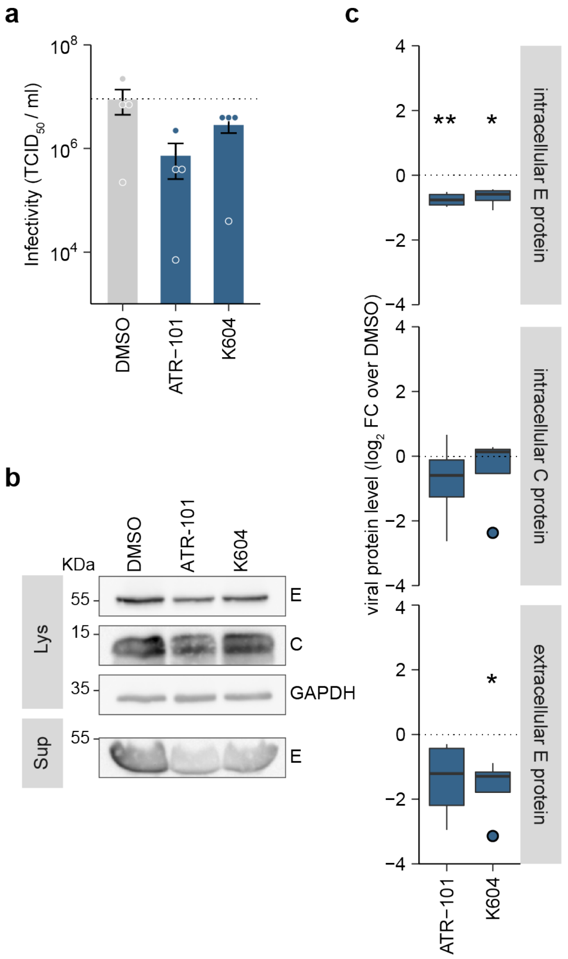

Supplementary Fig. 2: Post-infection treatment with SOAT1i reduces ZIKV infection in Hmc3 cells. (a–c) Hmc3 cells were infected with ZIKV (MOI 0.0025) for 1h. After removal of the virus inoculum, cells were treated with SOAT1i (9.72 µM ATR-101 or 20 µM K604) or DMSO. Lysates and supernatants were harvested at 48 hpi. Viral titers were determined by TCID$_{50}$ (mean ± SEM, n = 4, no asterisk = not significant, Welch´s t-test) (a). Cell lysates and supernatants were analyzed by immunoblotting using ZIKV C and orthoflavivirus E-protein specific antibodies (Lys = cell lysates, Sup = supernatant). GAPDH served as loading control. Shown is one representative immunoblot (b). Bands were quantified with ImageLab and intracellular signals were normalized to GAPDH. Box-and-whisker plot shows viral protein level as log$_2$ fold change over DMSO control (center line: median, box limits: upper and lower quartiles, whiskers: 1.5 x interquartile range, points: outliers, n = 4, **p* ≤ 0.05, ***p* ≤ 0.01, no asterisk = not significant, one-sample t-test). Individual data points represent outliers (c).

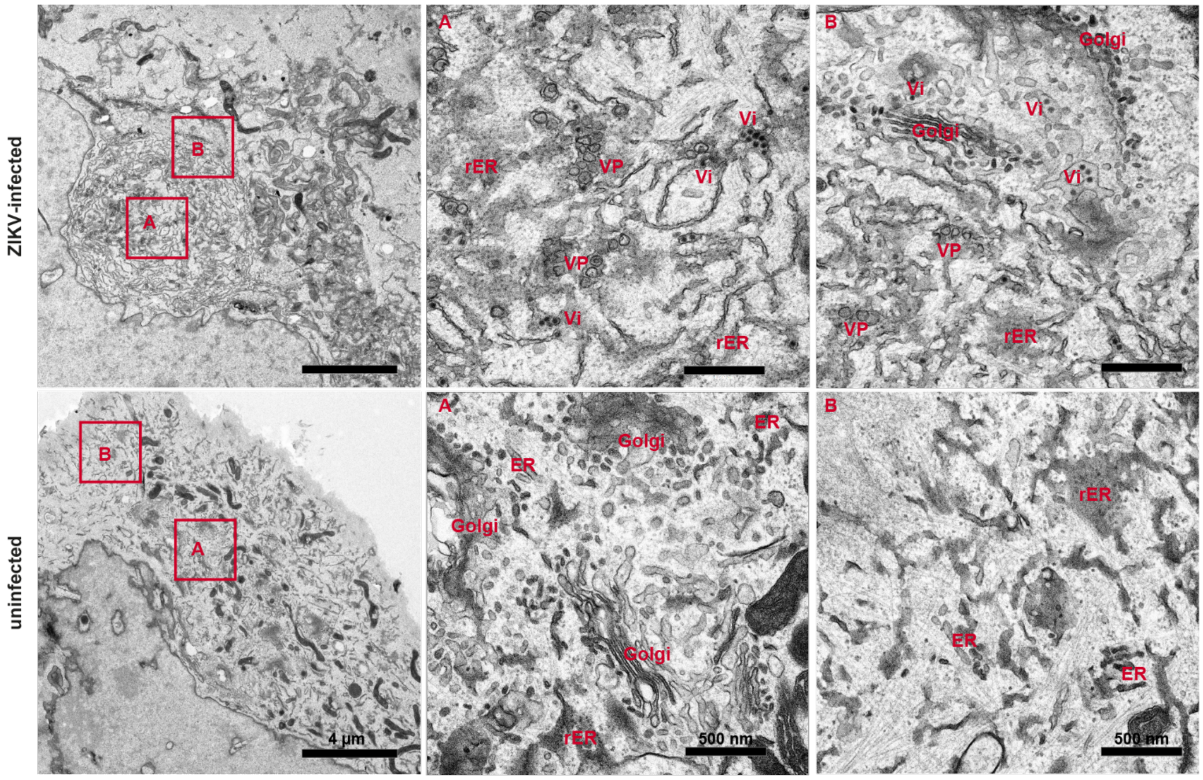

Supplementary Fig. 3: Transmission electron microscopy images of ZIKV-infected and uninfected Hmc3 cells. Hmc3 reporter cells were infected with ZIKV (MOI = 0.1), fixed at 24 hpi, and processed for EM. Shown are representative images of a ZIKV-infected and an uninfected cell (scale bars = 4 µm). Magnifications of the infected cell show viral structures (vesicle packets (VP) and virions (Vi)) that are absent in the uninfected cell (scale bars = 500 nm). rER = rough endoplasmic reticulum, ER = endoplasmic reticulum.

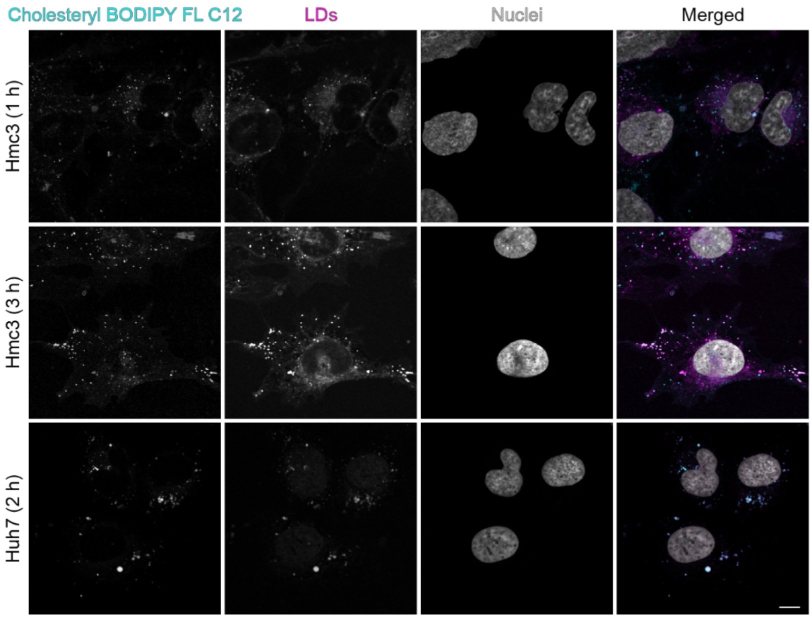

Supplementary Fig. 4: Uptake of fluorescently labelled cholesterol ester into Huh7 and Hmc3 cells. Hmc3 or Huh7 cells were treated with 1 µM Cholesteryl BODIPY FL C12 (cyan) in serum-free OptiMEM for the indicated period of time. Samples were fixed and LDs were visualized with BODIPY 655/676. Shown are representative images (scale bar 10 µm) (n = 1).

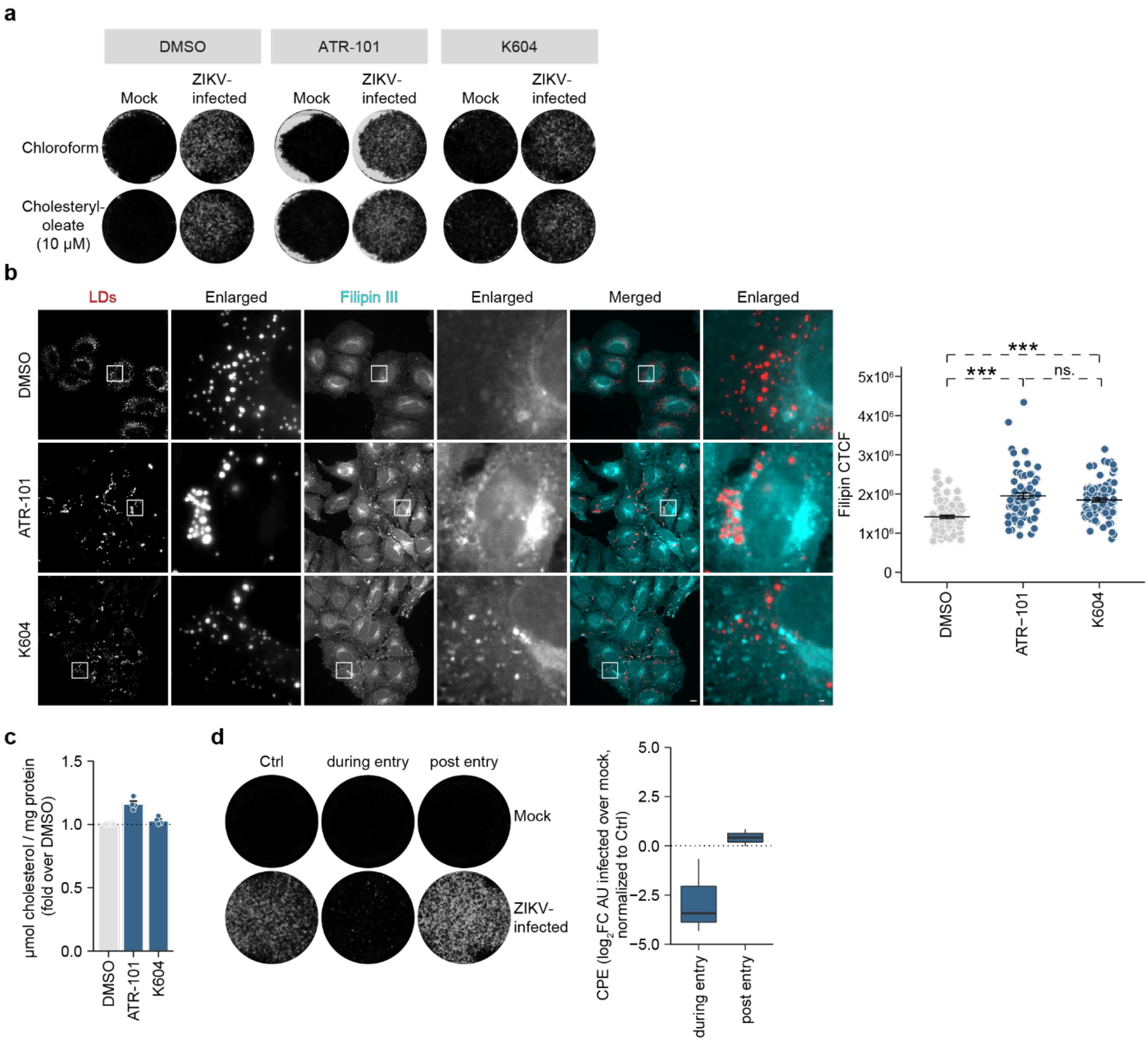

Supplementary Fig. 5: Free cholesterol accumulates in SOAT1i-treated Huh7 cells and impairs ZIKV infection. (a) Cells were cultured in serum-free OptiMEM containing DMSO, 20 µM K604, or 9.72 µM ATR-101 and 10 µM cholesteryl oleate or chloroform as vehicle control for 2 h before medium was changed to normal growth medium containing the respective inhibitors. After ZIKV infection (MOI 0.1) on the following day, cells were re-treated with cholesteryl oleate or chloroform as described, before medium was changed to normal growth medium with inhibitors for 3 d. Cells were fixed and CPE was visualized with crystal violet (n = 2). (b–c) SOAT1i treatment causes accumulation of free cholesterol. (b) Microscopic analysis of free cholesterol using filipin III. Huh7 cells were treated with either SOAT1i (9.72 µM ATR-101, 20 µM K604) or DMSO as vehicle control for 48 h before fixation. Staining was performed using LD540 and filipin III to visualize LDs and free cholesterol. Shown are representative images (scale bar 10 µm, scale bar inset 1 µm). The corrected total cell fluorescence (CTCF) for the filipin III channel was calculated for individual cells (# of cells n_{DMSO} = 90, $n_{ATR\text{-}101}$ = 75, n_{K604}= 84 from 2 independent experiments, mean ± SEM, *** $p \leq 0.001$, ns. = not significant, Welch´s t-test). (c) Cells were lysed 48 h post treatment with either SOAT1i (9.72 µM ATR-101, 20 µM K604) or DMSO as vehicle control and free cholesterol was measured. Depicted is the relative quantification of free cholesterol normalized to protein of 2 biological replicates from 2 independent experiments (mean ± SEM). (d) Cells were infected with ZIKV (MOI 0.1) or mock-infected and supplemented with 50 µg/ml water-soluble cholesterol (~40 mg of cholesterol per gram; balance methyl-β-cyclodextrin) during virus inoculation (during entry) or after infection (post entry). Cells were fixed after 3 d and stained with crystal violet to visualize surviving cells. CPE was quantified using Fiji. Box-and-whisker plot indicates CPE as $\log_2$ fold change over mock, normalized to the control (center line: median, box limits: upper and lower quartiles, whiskers: 1.5 x interquartile range, points: outliers, n = 3, no asterisk = not significant, one sample t-test).

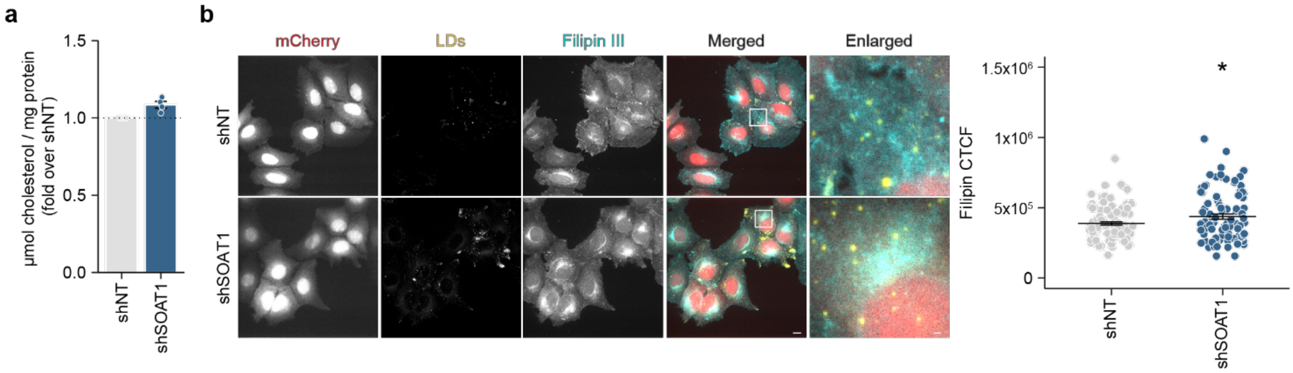

Supplementary Fig. 6: Free cholesterol accumulates in SOAT1 knockdown cells. (a) Huh7 cells were transduced with shRNA-expressing lentiviral particles, lysed at 5 dpt and free cholesterol was measured. Shown is the relative quantification of free cholesterol normalized to protein of 2 biological replicates from 2 independent experiments (mean ± SEM). (b) Knockdown cells were fixed at 5 or 6 dpt. LDs were visualized using BODIPY 493/503 and filipin III was used to stain free cholesterol. Shown are representative images (scale bar 10 µm, scale bar inset 1 µm). The CTCF for the filipin III channel was calculated for individual cells (# of cells n_{shNT} = 98, $n_{shSOAT1}$ = 95 from 3 independent experiments, mean ± SEM,* $p \leq 0.05$, Welch´s t-test).

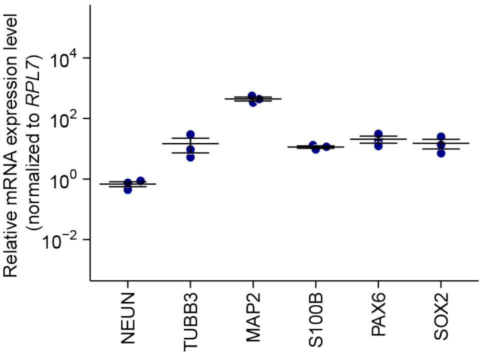

Supplementary Fig. 7: Marker gene expression in cerebral organoids. Total RNA was isolated from 3 individual cerebral organoids after 30–40 days of differentiation and maturation. mRNA expression level of the indicated neural markers were determined by qRT-PCR. Shown are relative mRNA expression levels ($2^{-\Delta CT}$) normalized to *RPL7*.

Supplementary Fig. 8: Uncropped immunoblots
Shown are the merged immunoblots of the colorimetric marker image and the chemiluminescent image for each antibody staining separately. On immunoblots with additional samples not shown in this manuscript, red squares indicate the relevant areas shown in main and supplemental figures.

PageRuler Prestained Protein Ladder (#26616 Thermo Fisher Scientific)

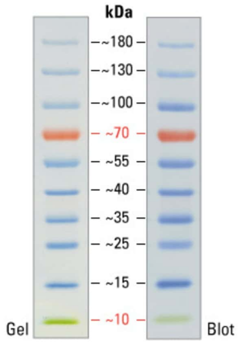

Figure 1b

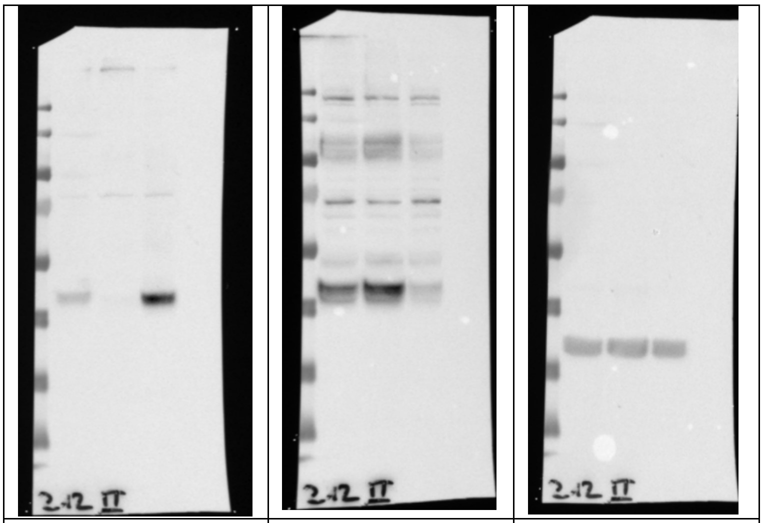

DGAT1	SOAT1	GAPDH

Figure 1d

Flavi E	GAPDH	
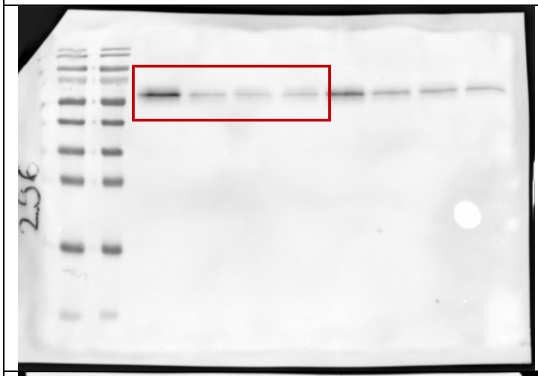	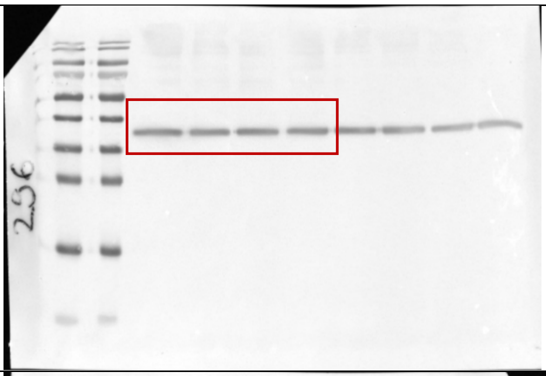	DENV
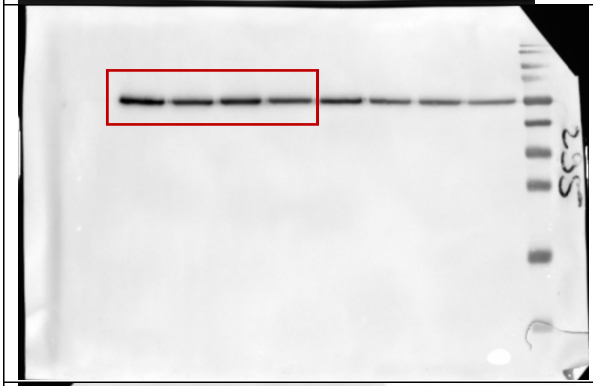	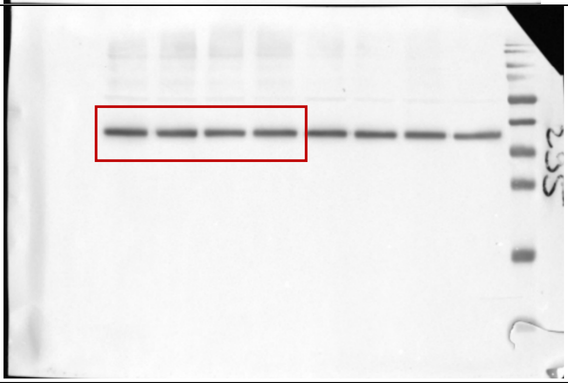	TBEV
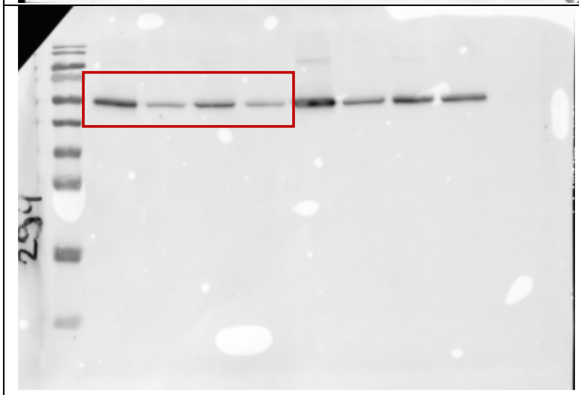	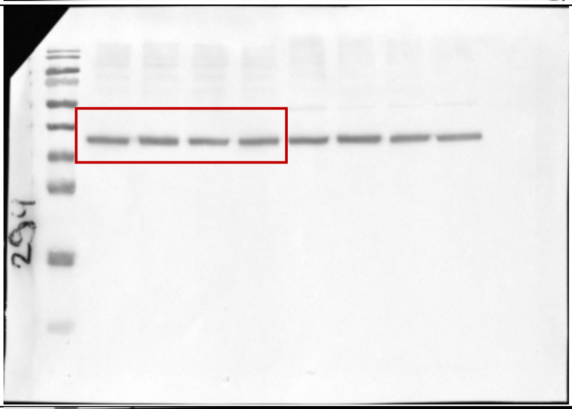	WNV
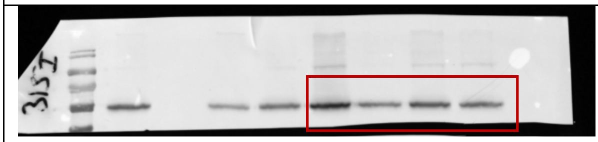	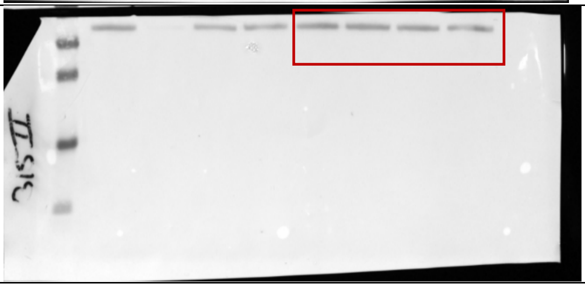	ZIKV

Figure 2c

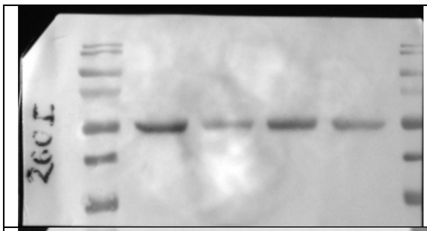	E protein
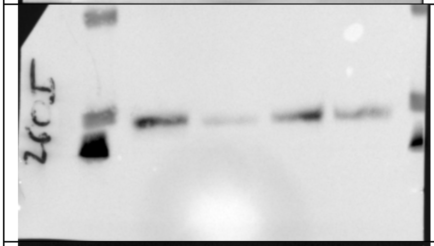	C protein
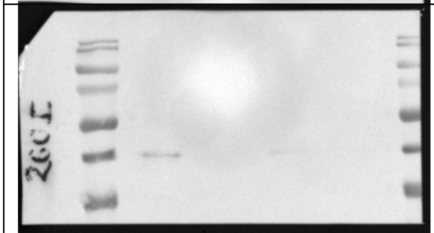	NS1 Protein
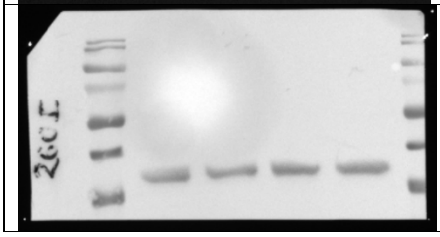	GAPDH

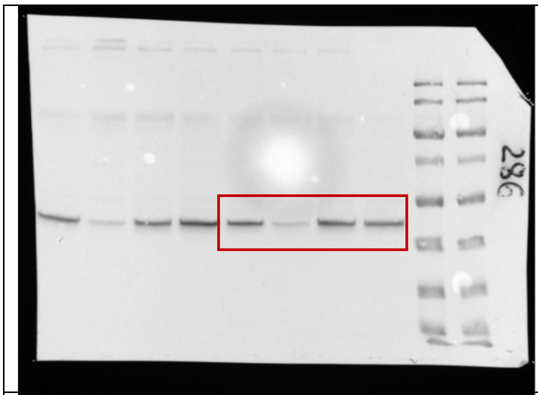	DGAT1
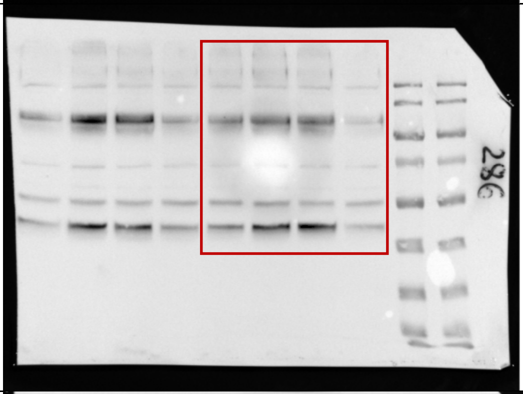	SOAT1
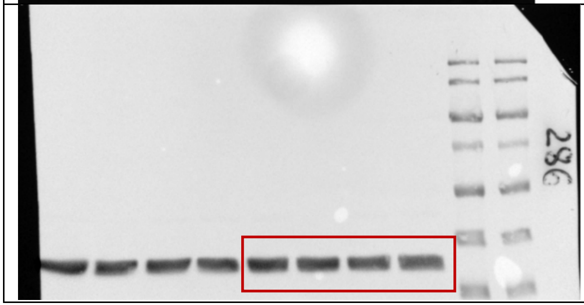	GAPDH

Figure 3b

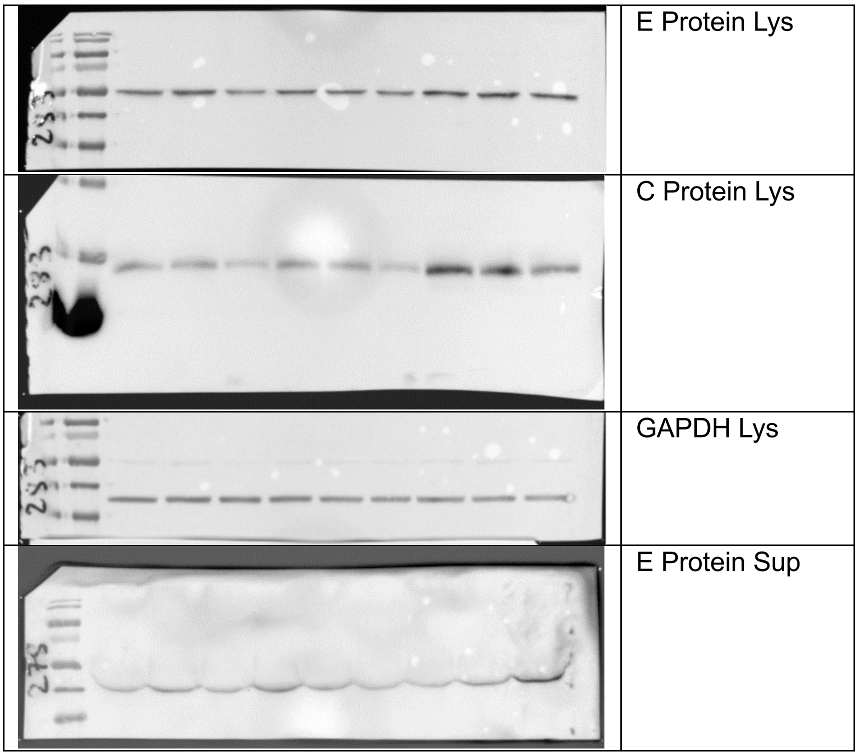

Figure 4a

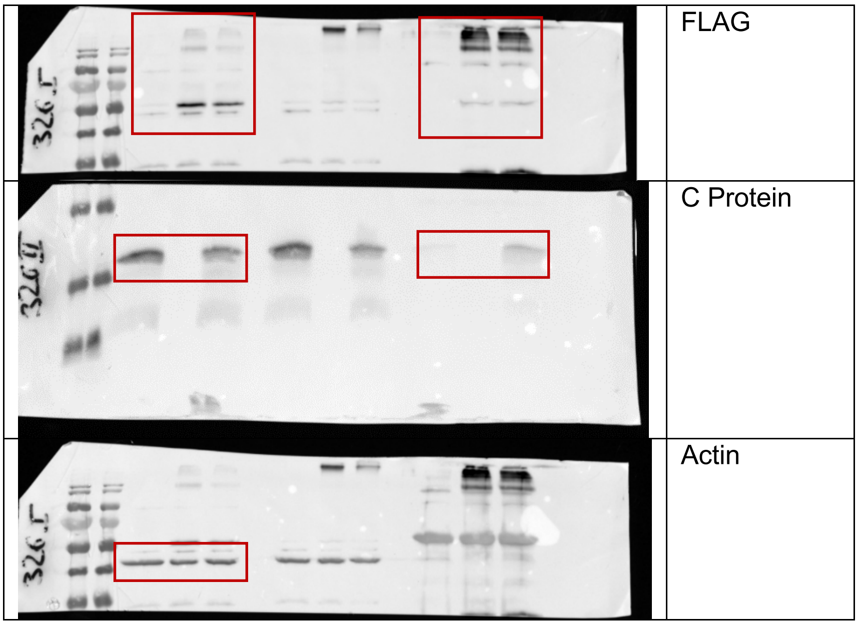

Figure 5b

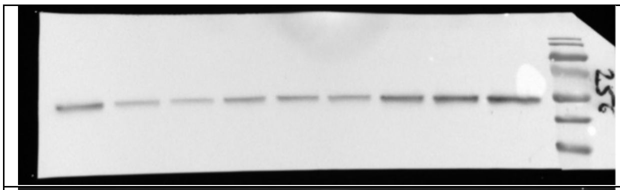	E Protein Lys
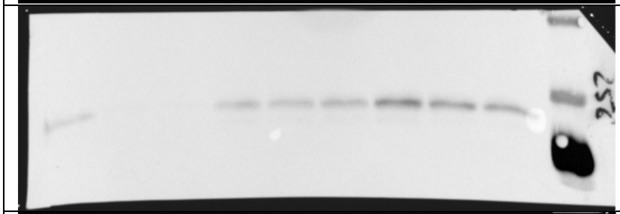	C Protein Lys
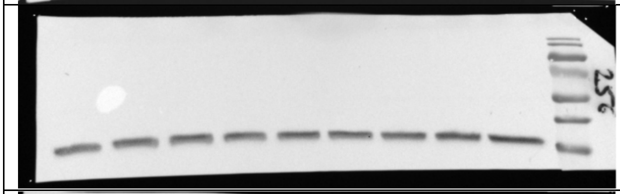	GAPDH Lys
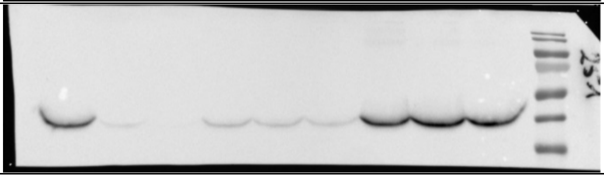	E Protein Sup

Figure 7b

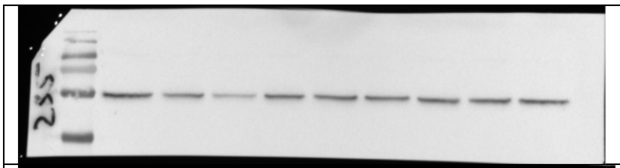	E Protein Lys
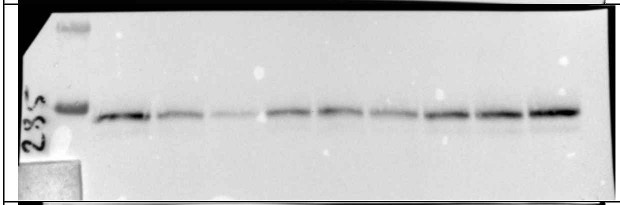	C Protein Lys
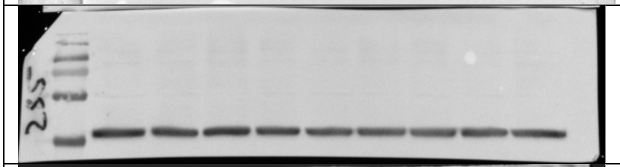	GAPDH Lys
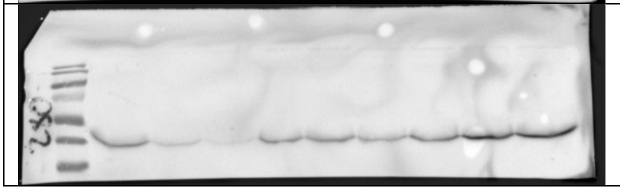	E Protein Sup

Figure 7d

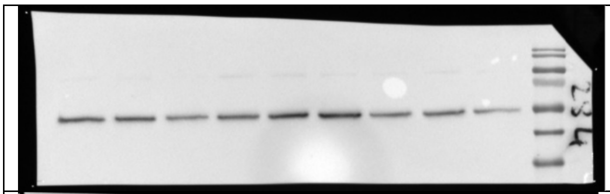	E Protein Lys
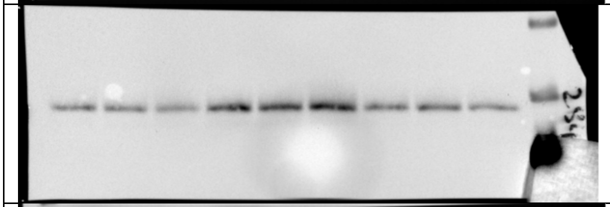	C Protein Lys
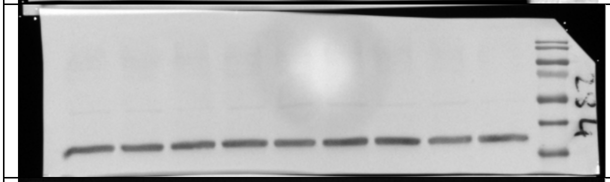	GAPDH Lys
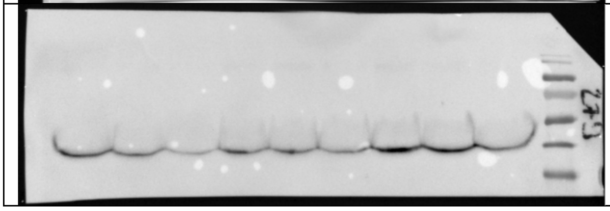	E Protein Sup

Figure 9d

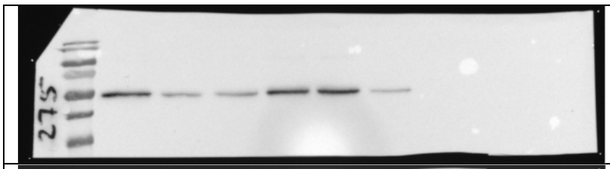	E Protein
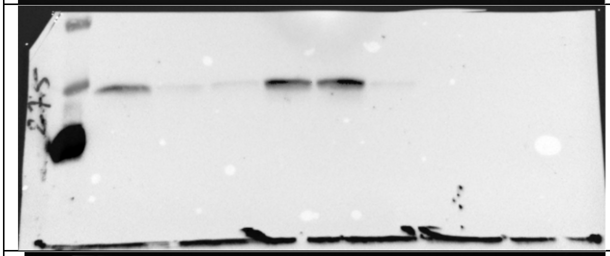	C Protein
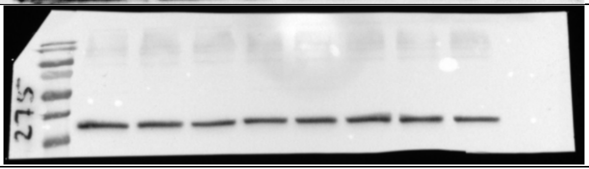	GAPDH

Supplementary Figure 1c

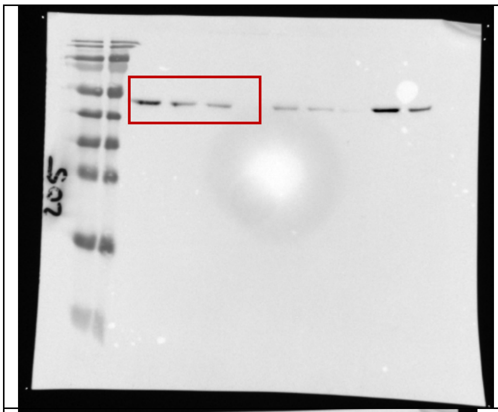	PLIN2
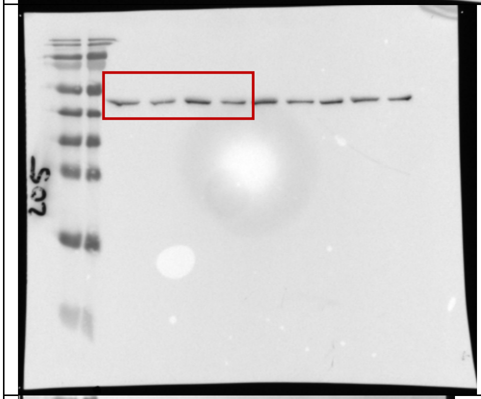	PLIN3
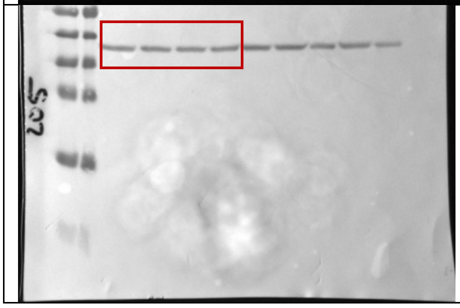	GAPDH

Supplementary Figure 2b

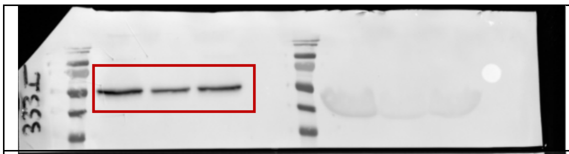	E Protein Lys
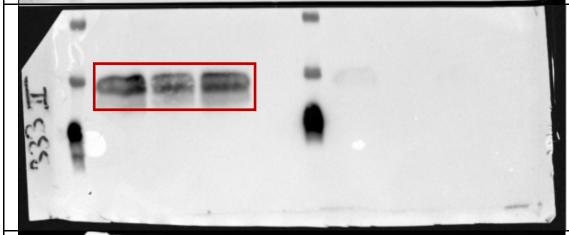	C Protein Lys
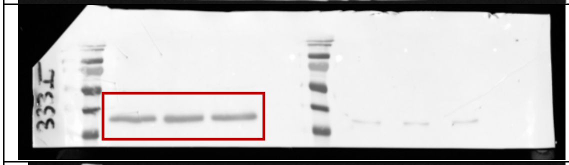	GAPDH Lys
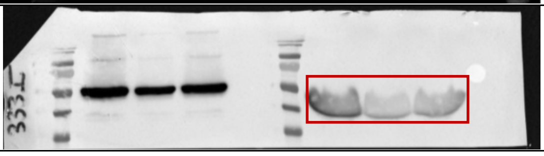	E Protein Sup
