# Supplementary material for: Inhibition of sterol O-acyltransferase 1 blocks Zika virus infection in cell lines and cerebral organoids
Source: Commun Biol. 2024 Sep 5;7:1089. doi: 10.1038/s42003-024-06776-4 (PMC11377701; doi:10.1038/s42003-024-06776-4)
Supplement: Supplementary file 5 — Reporting Summary [file 42003_2024_6776_MOESM5_ESM.pdf]

nature portfolio

Corresponding author(s): Eva Herker

Last updated by author(s): Aug 1, 2024

Reporting Summary

Nature Portfolio wishes to improve the reproducibility of the work that we publish. This form provides structure for consistency and transparency in reporting. For further information on Nature Portfolio policies, see our Editorial Policies and the Editorial Policy Checklist.

Statistics

For all statistical analyses, confirm that the following items are present in the figure legend, table legend, main text, or Methods section.

n/a	Confirmed	
☐	☒	The exact sample size (*n*) for each experimental group/condition, given as a discrete number and unit of measurement
☐	☒	A statement on whether measurements were taken from distinct samples or whether the same sample was measured repeatedly
☐	☒	The statistical test(s) used AND whether they are one- or two-sided<br>*Only common tests should be described solely by name; describe more complex techniques in the Methods section.*
☒	☐	A description of all covariates tested
☒	☐	A description of any assumptions or corrections, such as tests of normality and adjustment for multiple comparisons
☐	☒	A full description of the statistical parameters including central tendency (e.g. means) or other basic estimates (e.g. regression coefficient) AND variation (e.g. standard deviation) or associated estimates of uncertainty (e.g. confidence intervals)
☐	☒	For null hypothesis testing, the test statistic (e.g. *F*, *t*, *r*) with confidence intervals, effect sizes, degrees of freedom and *P* value noted<br>*Give P values as exact values whenever suitable.*
☒	☐	For Bayesian analysis, information on the choice of priors and Markov chain Monte Carlo settings
☒	☐	For hierarchical and complex designs, identification of the appropriate level for tests and full reporting of outcomes
☒	☐	Estimates of effect sizes (e.g. Cohen's *d*, Pearson's *r*), indicating how they were calculated

Our web collection on statistics for biologists contains articles on many of the points above.

Software and code

Policy information about availability of computer code

Data collection	Leica LAS X software for image acquisition<br>Chemidoc XRS documentation system (Biorad, Munich, Germany)<br>StepOne software V 2.1/Applied Biosystems for quantitative real-time PCR
Data analysis	RStudio Version 1.4.1717, R version 4.1.0: used to generate plots and statistical analysis. (Fiji is just) ImageJ 2.1.0/1.53c: used for microscopy image analysis and quantification. Code is available upon request.

For manuscripts utilizing custom algorithms or software that are central to the research but not yet described in published literature, software must be made available to editors and reviewers. We strongly encourage code deposition in a community repository (e.g. GitHub). See the Nature Portfolio guidelines for submitting code & software for further information.

Data

Policy information about availability of data

All manuscripts must include a data availability statement. This statement should provide the following information, where applicable:

- Accession codes, unique identifiers, or web links for publicly available datasets
- A description of any restrictions on data availability
- For clinical datasets or third party data, please ensure that the statement adheres to our policy

The raw data generated during this study are available from the lead contact upon request. The numerical source data for graphs and charts are shared in the Supplementary data.

Research involving human participants, their data, or biological material

Policy information about studies with human participants or human data. See also policy information about sex, gender (identity/presentation), and sexual orientation and race, ethnicity and racism.

Reporting on sex and gender	*Use the terms sex (biological attribute) and gender (shaped by social and cultural circumstances) carefully in order to avoid confusing both terms. Indicate if findings apply to only one sex or gender; describe whether sex and gender were considered in study design; whether sex and/or gender was determined based on self-reporting or assigned and methods used. Provide in the source data disaggregated sex and gender data, where this information has been collected, and if consent has been obtained for sharing of individual-level data; provide overall numbers in this Reporting Summary. Please state if this information has not been collected. Report sex- and gender-based analyses where performed, justify reasons for lack of sex- and gender-based analysis.*
Reporting on race, ethnicity, or other socially relevant groupings	*Please specify the socially constructed or socially relevant categorization variable(s) used in your manuscript and explain why they were used. Please note that such variables should not be used as proxies for other socially constructed/relevant variables (for example, race or ethnicity should not be used as a proxy for socioeconomic status). Provide clear definitions of the relevant terms used, how they were provided (by the participants/respondents, the researchers, or third parties), and the method(s) used to classify people into the different categories (e.g. self-report, census or administrative data, social media data, etc.) Please provide details about how you controlled for confounding variables in your analyses.*
Population characteristics	*Describe the covariate-relevant population characteristics of the human research participants (e.g. age, genotypic information, past and current diagnosis and treatment categories). If you filled out the behavioural & social sciences study design questions and have nothing to add here, write "See above."*
Recruitment	*Describe how participants were recruited. Outline any potential self-selection bias or other biases that may be present and how these are likely to impact results.*
Ethics oversight	*Identify the organization(s) that approved the study protocol.*

Note that full information on the approval of the study protocol must also be provided in the manuscript.

Field-specific reporting

Please select the one below that is the best fit for your research. If you are not sure, read the appropriate sections before making your selection.

☒ Life sciences ☐ Behavioural & social sciences ☐ Ecological, evolutionary & environmental sciences

For a reference copy of the document with all sections, see nature.com/documents/nr-reporting-summary-flat.pdf

Life sciences study design

All studies must disclose on these points even when the disclosure is negative.

Sample size	No sample size-calculation was performed. In general, a minimum of 3 independent experiments were performed to enable statistical analysis. In case of microscopy data analysis, more than 15 individual cells from different experiments were analyzed, a number that is sufficient to describe the respective phenotypes. The exact sample sizes are stated in the Figure Legends.
Data exclusions	Data were only excluded when the quality of the samples was not sufficient (i.e., deviating house keeping gene expression); for immunofluorescence image analysis, only cells with an intact nucleus were used for quantification.
Replication	In general, 3 or more independent experiments were performed in order to verify reproducibility. The number of independent experiments is stated in the Figure Legends. All replication attempts were successful.
Randomization	No specific method of randomization was used for generation of samples as they were performed on uniform biological material.
Blinding	The investigators were not blinded throughout the study. Blinding was not possible as the experiments were performed by individual

Blinding | investigators who were aware of the experimental groups.

Reporting for specific materials, systems and methods

We require information from authors about some types of materials, experimental systems and methods used in many studies. Here, indicate whether each material, system or method listed is relevant to your study. If you are not sure if a list item applies to your research, read the appropriate section before selecting a response.

Materials & experimental systems

n/a	Involved in the study
☐	☒ Antibodies
☐	☒ Eukaryotic cell lines
☒	☐ Palaeontology and archaeology
☒	☐ Animals and other organisms
☒	☐ Clinical data
☒	☐ Dual use research of concern
☒	☐ Plants

Methods

n/a	Involved in the study
☒	☐ ChIP-seq
☒	☐ Flow cytometry
☒	☐ MRI-based neuroimaging

Antibodies

Antibodies used

Flavivirus group antigen antibody (D1-4G2-4-15 (4G2), NBP2-52666, Novus Biologicals), Flavivirus NS1 antibody ((D/2/D6/B7), ab214337, abcam), Zika virus capsid protein antibody (GTX133317), SOAT1 antibody (GTX32890) (all GeneTex), GAPDH antibody (clone G-9, sc-365062), DGAT1 antibody (clone H-255, sc-32861) (all Santa Cruz Biotechnology), J2 dsRNA antibody (Scicons/Jena Biosciences 314 RNT-SCI-10010200), PLIN2 antibody (610102, Progen), PLIN3 antibody (HPA006427, Sigma) HRP-labelled secondary antibodies (Jackson ImmunoResearch), Anti-Rabbit IgG HRP conjugated (Rabbit TrueBlot) (clone eB182, 18-8816-33, Rockland Immunochemicals), Alexa647-conjugated secondary antibody (donkey, IgG (H+L), A-31571, Invitrogen), Strep-tag II antibody (ab184224, abcam), FLAG antibody (F7425, Sigma), beta-actin antibody (clone AC-74, A2228-200µl, Sigma)

Validation

SOAT1 and DGAT1 antibody specificity was confirmed by immunoblotting with the respective shRNA samples.
Flavivirus group antigen antibody (D1-4G2-4-15 (4G2), NBP2-52666, Novus Biologicals), Flavivirus NS1 antibody ((D/2/D6/B7), ab214337, abcam), and Zika virus capsid protein antibody (GTX133317) specificity in immunoblot analysis was confirmed using mock-infected cells as control.
PLIN2 and PLIN3 antibodies were validated before (Lassen et al., 2019 PMID 30559250).
J2 dsRNA antibody was validated before (Herker et al., 2010 PMID 20935628) and mock-infected cells were used as control for immunofluorescence (not shown in the manuscript).
GAPDH and beta-actin antibodies shows a distinct signal at the expected molecular weight in immunoblot analysis.
Strep-tag II antibody has been tested by the manufacturer and the specificity of the signal in immunofluorescence was confirmed by using untransfected cells as control (https://www.abcam.com/en-de/products/primary-antibodies/strep-tag-ii-antibody-gt661-ab184224#)
FLAG antibody specificity was confirmed in immunoblot analysis using non-FLAG-expressing cells as control.

Eukaryotic cell lines

Policy information about cell lines and Sex and Gender in Research

Cell line source(s)

HEK293T: American Type Culture Collection
VeroE6: American Type Culture Collection
Huh7.5 cells: Apath, LCC
1321N1 cell line (86030402): purchased from Merck
SH-SY5Y cell line (94030304): purchased from Merck
Huh7 cells provided by Ralf Bartenschlager
BHK-21 cells provided by César Muñoz-Fontela
Hmc3 cells provided by Alexander Slowik
HMGU#1 human induced pluripotent stem cells (hiPSCs): Pluripotent Stem Cell core facility at the Helmholtz Institute, Munich, Germany
TISSUi006A hiPSCs: organoid platform at the Max-Delbrück Institute, Berlin, Germany
Both iPSC cell lines originate from a male donor and registered in the Human Pluripotent Stem Cell Registry (https://hpscreg.eu). The code for the TISSUi006A is CVCL_WU61 for the HMGU#1 is CVCL_YT30

Authentication

HEK293T, VeroE6, Huh7.5, Huh7, BHK-21, Hmc3, and 1321N1 were authenticated by STR profiling, all other cell lines were recently aquired and not authenticated by us.

Mycoplasma contamination

Cell lines were regularly tested for mycoplasma contamination.

Commonly misidentified lines
(See ICLAC register)

Cell lines used are not in ICLAC.

Plants

Seed stocks	*Report on the source of all seed stocks or other plant material used. If applicable, state the seed stock centre and catalogue number. If plant specimens were collected from the field, describe the collection location, date and sampling procedures.*
Novel plant genotypes	*Describe the methods by which all novel plant genotypes were produced. This includes those generated by transgenic approaches, gene editing, chemical/radiation-based mutagenesis and hybridization. For transgenic lines, describe the transformation method, the number of independent lines analyzed and the generation upon which experiments were performed. For gene-edited lines, describe the editor used, the endogenous sequence targeted for editing, the targeting guide RNA sequence (if applicable) and how the editor was applied.*
Authentication	*Describe any authentication procedures for each seed stock used or novel genotype generated. Describe any experiments used to assess the effect of a mutation and, where applicable, how potential secondary effects (e.g. second site T-DNA insertions, mosiacism, off-target gene editing) were examined.*
